# A prime-boost immunization with rBCG expressing HIV-1 Gag, RT and gp120 and SAAVI MVA-C elicits immune responses in blood and MALT of rhesus macaques

**DOI:** 10.1186/1742-4690-9-S2-P40

**Published:** 2012-09-13

**Authors:** GK Chege, R Chapman, EG Shephard, A Williamson

**Affiliations:** 1 University of Cape Town, Cape Town, South Africa

## Background

BCG pantothenate auxtroph (ΔpanCD) is safer to use as a live vaccine vector than wild-type BCG. We constructed 3 recombinant BCGΔpanCD candidate vaccines expressing HIV-1 subtype C Gag, RT and Env (gp120). The current study investigated immune responses in rhesus macaques following a prime with a mixture of these rBCG vaccines and a boost with SAAVI MVA-C (MVA).

## Methods

Chinese rhesus macaques (n=8) were primed twice with a mixture of rBCG, 12 weeks apart. A control group (n=4) was mock-primed with a control BCG. Both groups were boosted with MVA. Two weeks after the MVA vaccination, two macaques from the rBCG-primed group were euthanased and jejunum, spleen and inguinal, mesenteric, iliac and bronchial lymph nodes were harvested for isolation of mononuclear cells. HIV-1-specific IFN-gamma ELISPOT responses were measured in the blood and these tissues using pools of overlapping HIV-1 peptides.

## Results

Vaccination with rBCG elicited modest HIV-specific responses in the blood in 5 of 8 animals, 4 of which responded after the first rBCG vaccination. These responses were to either Env or to both Env and Gag proteins and the cumulative responses ranged from 50 to 172 sfu/10^6^ PBMC. After boosting with MVA, HIV-specific responses were detected in 6 of the 8 animals (mean: 932±1100 sfu/10^6^ PBMC). These responses were directed to Gag, RT, and Env proteins but not Nef or Tat. No responses were detected in the control animals before or after MVA vaccination. At necropsy, HIV-specific responses were detected in the peripheral blood, spleen, inguinal, iliac and bronchial lymph nodes of 1 of 2 animals. The cumulative responses ranged from 112 to 714 sfu/10^6^ cell input.

## Conclusion

These data demonstrate that our rBCGΔpanCD candidate vaccines, when given in a prime-boost combination with SAAVI MVA-C, induce vaccine-specific immune responses in both the peripheral blood and MALT.

